# Real-Time Monitoring of SO_2_ Emissions Using a UV Camera with Built-in NO_2_ and Aerosol Corrections

**DOI:** 10.3390/s22103900

**Published:** 2022-05-20

**Authors:** Yuanhui Xiong, Kuijun Wu, Guangbao Yu, Zhenwei Chen, Linmei Liu, Faquan Li

**Affiliations:** 1 State Key Laboratory of Magnetic Resonance and Atomic and Molecular Physics, Innovation Academy for Precision Measurement Science and Technology, Chinese Academy of Sciences, Wuhan 430071, China; xiongyuanhui@apm.ac.cn (Y.X.); yuguangbao@apm.ac.cn (G.Y.); chenzhenwei@apm.ac.cn (Z.C.); liulinmei@apm.ac.cn (L.L.); 2 University of Chinese Academy of Sciences, Beijing 100049, China; 3 School of Opto-Electronic Information Science and Technology, Yantai University, Yantai 264005, China; wukuijun@ytu.edu.cn

**Keywords:** SO_2_ camera, UV imaging, NO_2_ absorption correction, aerosol scattering, remote sensing, DOAS

## Abstract

Nitrogen dioxide (NO_2_) absorption correction of the sulfur dioxide (SO_2_) camera was demonstrated for the first time. The key to improving the measurement accuracy is to combine a differential optical absorption spectroscopy (DOAS) instrument with the SO_2_ camera for the real-time NO_2_ absorption correction and aerosol scattering correction. This method performs NO_2_ absorption correction by the correlation between the NO_2_ column density measurement of the DOAS and the NO_2_ optical depth of the corresponding channel from the SO_2_ camera at a narrow wavelength window around 310 and 310 nm. The error of correction method is estimated through comparison with only using the second channel of the traditional SO_2_ camera to correct for aerosol scattering and it can be reduced by 11.3% after NO_2_ absorption corrections. We validate the correction method through experiments and demonstrate it to be of greatly improved accuracy. The result shows that the ultraviolet (UV) SO_2_ camera system with NO_2_ absorption corrections appears to have great application prospects as a technology for visualized real-time monitoring of SO_2_ emissions.

## 1. Introduction

Emissions of polluting gases from industries and ships have brought severe air pollution, particularly in developed and coastal areas. Atmospheric pollutants from industries and ships are mainly generated from fuel combustion. The main product of combustion includes sulfur dioxide (SO_2_), nitrogen dioxide (NO_2_), carbon dioxide (CO_2_), and particles [1,2,3]. SO_2_ is of special importance and interest. As a toxic gas, SO_2_ is responsible for many deleterious effects on human health, the environment, and the climate. SO_2_ emissions contribute to the formation of sulfate aerosols and small particles, which may penetrate deeply into the lungs, and, in sufficient quantity, can contribute to health problems. Increased SO_2_ in the Earth’s atmosphere can alter the radiation balance by intercepting scattered light. However, its far distance and low concentration make SO_2_ emissions difficult to measure by using the existing techniques.

The existing optical techniques for SO_2_ concentration measurements are an effective tool for high concentrations or fixed sources or near distances. Raman scattering lidar and differential absorption lidar are active detection methods [4,5] that can realize stereoscopic detection of pollutant space and have distance resolution capability. However, their spatial resolution is low and cannot meet the requirements of portable and mobile applications. For the sake of portability and engineering, passive detection methods have emerged. Fourier transform infrared spectroscopy and Fourier function analysis are characterized by multi-component analysis, wide measurement range, and fast analysis speed [6,7,8]. Differential optical absorption spectroscopy (DOAS) provides fast response and real-time monitoring to characterize and obtain data [9,10,11]. Recent advancements in spectroscopic techniques allow remote analysis of many species. Non-imaging measurement techniques allow us to measure the total column density of the trace gas along a single direction within the plume. Imaging DOAS (I-DOAS) combines the advantages of DOAS with imaging capabilities [12,13]. This technique allows the spectroscopic measurement of 2D pollutant gas distributions and improves the accuracy of quantitative measurement. A drawback of scanning DOAS systems is a relatively long time to acquire a 2D image of the trace gas distribution.

The development of remote sensing techniques based on a novel ultraviolet (UV) SO_2_ camera system [14] for trace gas measurement has emerged. The SO_2_ camera is increasingly used in SO_2_ emission due to its ability to remotely measure the 2D distribution of SO_2_ path concentrations in space and the emission rate over time in real-time. The measurement of 2D SO_2_ distribution in volcanic plumes has been realized by using a single UV camera. UV cameras have been increasingly established [15,16]. The University of Heidelberg discussed the theoretical basis of UV cameras and calibration issues, including the measurement principle, data evaluation, and solar zenith angle [17]. They used the 3D backward Monte Carlo radiative transfer model to describe the spectral radiance transmitted through the filter [18]. Christoph Kern et al. used seven different UV cameras and four different filters to continuously monitor the volcanic plumes in real-time [19]. Osorio, M et al. proposed a new two-image method (2-IM) to acquire background images and quantify SO_2_ emissions from industrial sources [20]. However, UV cameras are mostly used to measure higher concentrations of SO_2_ emissions from volcanic sources. Few measurements of emissions with low concentrations from industries and ships have been performed with UV cameras. Some persistent problems may arise when UV camera systems are used to measure the SO_2_ emissions of industrial and ship sources.

UV SO_2_ cameras are used to measure the SO_2_ absorption in a narrow wavelength (310 nm), and the effect of aerosol scattering can be partially corrected by measuring the weak absorption of SO_2_ around 330 nm. The measured signal is the integral intensity of the incident spectrum over the filter transmittance window. SO_2_ cameras are certainly limited by the fact that only one trace gas can be measured and the other spectral interferences and the effect of aerosol scattering on the particulate matter can potentially lead to an inaccurate measurement.

This work aims to correct NO_2_ absorption for real-time measurement of SO_2_ emissions with UV cameras. This proposed method performs NO_2_ absorption correction by the correlation between the NO_2_ column density measurement of the DOAS and the NO_2_ optical depth of the corresponding channel from the SO_2_ camera at a narrow wavelength window around 310 and 310 nm. Theoretical simulation analysis shows that different NO_2_ column densities have a relatively large influence on the error of SO_2_ column density measured by the UV camera. In this paper, we briefly described the theoretical basis for pertinent aspects of spectral consideration. The basic principles of UV SO_2_ camera methodology and the simulation of error analysis are described. The main experimental results are obtained from a plant in Wuhan. In order to further enhance the measurement accuracy, the Mie scattering corrections are made. The following discusses the measurement uncertainty of SO_2_ column density. Then, we show the SO_2_ error on NO_2_ absorption corrections.

## 2. Spectral Consideration

**Figure 1:**
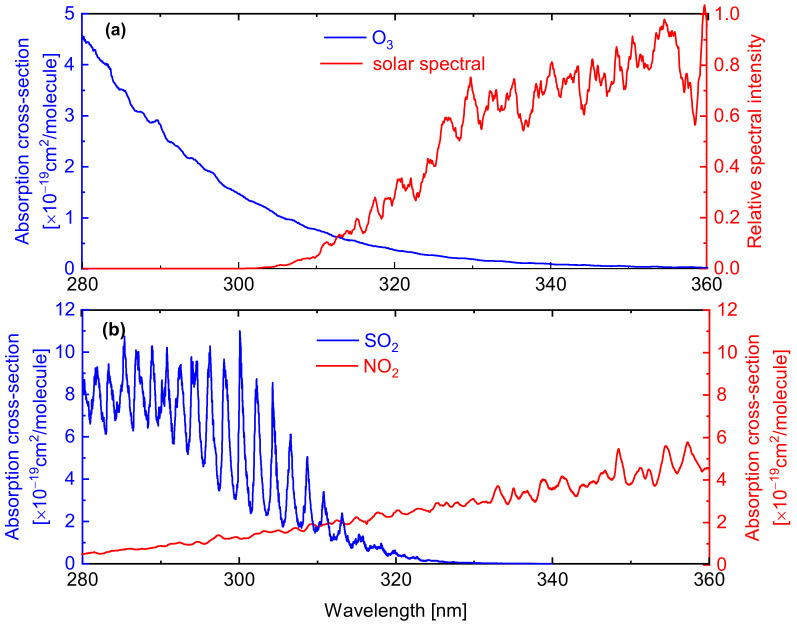
(**a**) Scattered solar relative spectral intensity varying with wavelength (red line) and absorption cross-section of O_3_ varying with wavelength (blue line); (**b**) Absorption cross-section of SO_2_ (blue line) and absorption cross-section of NO_2_ (red line), which are calculated on HITRAN.

When scattered solar radiation passes through industrial and ship emission plumes, light is scattered and absorbed along the path. This condition is caused by absorption within the band due to other gases (such as NO_2_, O_3_), scattering of particles, and multiple scattering. In the UV region, the O_3_ absorption [blue line in Figure 1a] blocks part of the scattered solar spectra and the UV cannot break through the ozone at a wavelength shorter than 300 nm. In Figure 1a (red line), the relative scattered solar spectral intensity reaches the ground at a spectral resolution of 0.1 nm, which is calculated on MODTRAN [21]. The UV cameras are used to measure the SO_2_ emissions in a narrow wavelength band centered at 310 nm, which is the region of the scattered solar spectral intensity with an increasing magnitude as the wavelength increases. The spectral interference from other major emission products (such as NO_2_) should be considered for measuring SO_2_ emissions. The measurement of SO_2_ emission from industries and ships cannot be ignored due to the extremely high NO_2_ absorption interference. Figure 1b shows the SO_2_ absorption spectrum (blue line) and NO_2_ absorption spectrum (red line) in the spectral range of 280–360 nm.

As shown in Figure 1b, the absorption band of SO_2_ overlaps with the absorption band of NO_2_, and the absorption cross-sections of SO_2_ and NO_2_ are comparable in the chosen spectral window. We calculated the SO_2_ column density (CD) error caused by the interference of NO_2_ absorption. In accordance with the Beer–Lambert law to resolve the SO_2_ signal, the signal channel and the reference channel can be expressed as:

(1) $$I(\lambda_{on},\lambda_{off})=I_{0}(\lambda_{on})\cdot exp\left[-(\sigma_{SO_{2}}(\lambda_{on},\lambda_{off})\cdot S_{SO_{2}})-(\sigma_{NO_{2}}(\lambda_{on},\lambda_{off})\cdot S_{NO_{2}})-\tau_{Mie}(\lambda_{on},\lambda_{off})\right]$$

where the incident spectral radiation intensity I and radiation intensity $I_{0}$ are the intensity images after and before traversing the plume at wavelength λ, $\sigma_{SO_{2}}(\lambda_{on})$ is the absorption cross-section of SO_2_, $S_{SO_{2}}$ is the SO_2_ CD or the integral of SO_2_ concentration along the effective optical path, $\sigma_{NO_{2}}(\lambda_{on})$ is the absorption cross-section of NO_2_, and $S_{NO_{2}}$ is its CD. $\tau_{SO_{2}}$ is the apparent absorbance, which is the difference between the optical depth (OD) for $\lambda_{on}$ and $\lambda_{off}$. No aerosols are assumed to be present in the emission plume. We obtain an expression for the SO_2_ optical depth:

(2) $$\tau_{SO_{2}}=\tau_{310}-\tau_{330}=\ln\left(\frac{I(\lambda_{on})}{I_{0}(\lambda_{on})}\right)-k_{NO_{2}}\cdot \ln\left(\frac{I(\lambda_{off})}{I_{0}(\lambda_{off})}\right)$$

where $\kappa_{NO_{2}}=\tau_{NO_{2}}(\lambda_{on})/\tau_{NO_{2}}(\lambda_{off})=\sigma_{NO_{2}}(\lambda_{on})/\sigma_{NO_{2}}(\lambda_{off})$ is the ratio between the integral of NO_2_ absorption cross-section at two region wavelengths of narrow-band filters. In this work, $\lambda_{on}$ is 310 nm, and $\lambda_{off}$ is 330 nm with narrow-band filters in the center wavelength.

**Figure 2:**
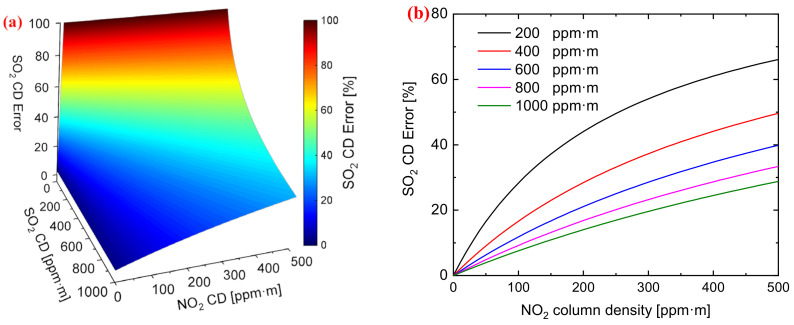
(**a**) Result of SO_2_ column density (CD) error under the influence of NO_2_ CD; (**b**) Error varying with NO_2_ CD for five different SO_2_ CDs (200 ppm⋅m, 400 ppm⋅m, 600 ppm⋅m, 800 ppm⋅m, and 1000 ppm⋅m ).

Figure 2 shows the simulated SO_2_ CD measurement error under the influence of NO_2_ CD. With the significant increase in NO_2_ CD towards high concentration, the signal channel ($\lambda_{on}$) SO_2_ OD is particularly influenced by the NO_2_ CD. This error is the main source for measuring SO_2_ emissions. It is important to eliminate the influence of NO_2_ absorption in the plume.

Figure 2b shows the calculated error varying with the NO_2_ CD at different SO_2_ CDs. The error is approximately the monotone function of NO_2_ CD, and the relation between them is particularly affected by the NO_2_ CD. The error clearly increases with the increase in NO_2_ CD. Therefore, the error of measuring SO_2_ CD can be effectively eliminated if the ratio of the two NO_2_ ODs of two channels is known.

## 3. Methodology

### 3.1. Experiment Instrument

**Figure 3:**
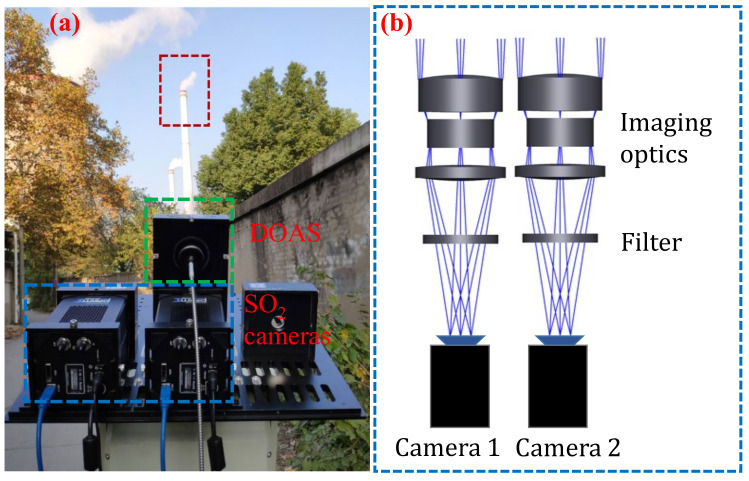
(**a**) Photograph of the SO_2_ cameras system and differential optical absorption spectroscopy (DOAS) system; (**b**) The optical diagram of the SO_2_ camera system.

To verify the accuracy of the theoretical analysis and simulation, we used SO_2_ cameras to acquire images of SO_2_ emissions from the plant near Wuhan on 16 July 2021. The SO_2_ camera system is shown in Figure 3. Figure 3a shows a photograph of the SO_2_ camera system and the DOAS system. The main part of the instrument (see green dotted line area in Figure 3a) is the DOAS system, a lens, and spectrometer-connected fiber. As well as two cameras (blue dotted line area in Figure 3a), each with a lens and a bandpass filter (310 nm and 330 nm). From outside the plant, we can observe one stack from which SO_2_ emissions are monitored (red dotted line area in Figure 3a). Figure 3b shows a schematic diagram of the SO_2_ camera system (correspond to the blue dotted line in Figure 3a). The SO_2_ cameras (Prime 95B Blue) used for these experiments have the most sensitive scientific back-illuminated CMOS sensor (1200 × 1200 pixels, 11 μm × 11 μm pixel size) manufactured by Photometrics (Tucson, AZ, USA). The quantum efficiency of approximately 50–55% at 310 and 330 nm of each SO_2_ camera is high from 200–1000 nm. The UV lens (UV1054B) of 105 mm focal length (Universe Kogaku America Inc., Oyster Bay, NY, USA) with a total field of view (FOV) of 9.8° and F-number of 4.0 were mounted in front of each camera. Two UV narrow-band filters (ASAHI SPECTRA, Torrance, CA, USA), with a central wavelength of 310 (XBPA310) and 330 nm (XBPA330) and full width at half maximum of 10 nm, were used for the measurements. Two narrow-band filters were mounted between the lens and the cameras. This setup was chosen to reduce the influences from the illumination angle. Two cameras were fixed side by side to simultaneously capture the current polluting gases of industrial emissions by using a function generator (Tektronix AFG 3022B, Beaverton, OR, USA) with a frequency of 1–5 Hz. For comparison, a DOAS system was installed on the two UV cameras. The DOAS system consisted of an Ocean Optics Maya (Dunedin, FL, USA) 2000Pro spectrometer with a spectral range between 247 and 390 nm and a resolution of 0.035 nm, a 600 μm optical fiber, and a telescope with the same quartz lens as the UV camera.

**Figure 4:**
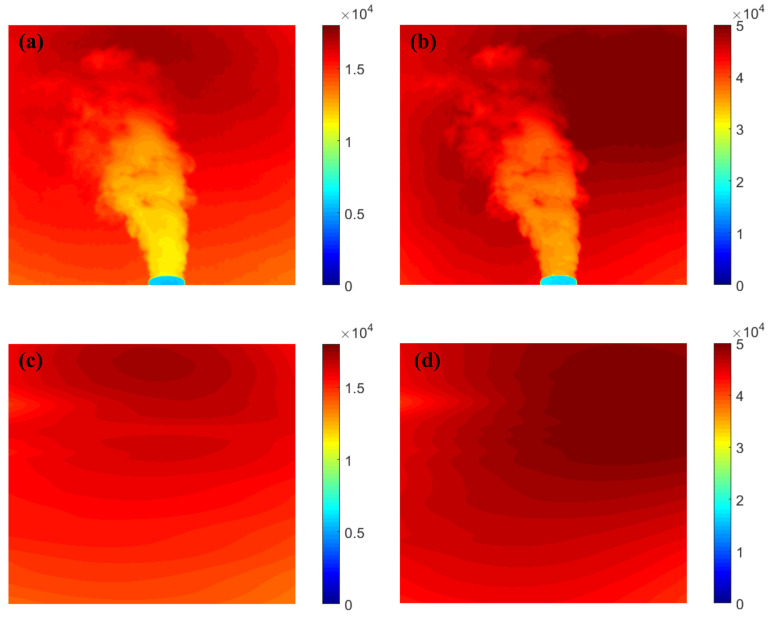
Example of raw images at $\lambda_{on}=310$ nm (**a**); and at $\lambda_{off}=330$ nm (**b**); and the artificial background sky images at $\lambda_{on}=310$ nm (**c**); and at $\lambda_{off}=330$ nm (**d**).

The SO_2_ camera system is portable. The location (approximately 500 m from the plant through a laser rangefinder) was chosen to be as close as possible to the plant plume emissions (red dotted line area in Figure 3a) to reduce light dilution effects. The exposure times (approximately 0.1 s for 310 nm and 0.06 s for 330 nm) were used to acquire images at approximately 50–60% of the intensity saturation level. The camera with a 310 nm narrow-band filter required long exposure times due to the low intensity of solar radiation. The UV cameras and DOAS system were controlled through panning and tilting. The exposure time of the camera system should be multiple times, especially for the measurement of industrial emissions, to overcome the mechanical complexity of the camera system and the influence of the zenith angle. The images were captured with acquisition rates of 5 Hz, with an additional collection of dark images prior to the capturing of plume sequences. The acquisition and subtraction of a dark frame are required to correct the dark current and electronic noise on the UV camera system. Figure 4a,b shows a pair of the raw images ($\lambda_{on}$ and $\lambda_{off}$) of an industrial plume. The raw images were processed following the protocols, which are already described in the literature [20], including the subtraction of a dark current and image matching. Simultaneously, the background sky images ($\lambda_{on}$ and $\lambda_{off}$) of the plume can be constructed from the raw plume images by plume segmentation and interpolation. Figure 4c,d shows a pair of the generated artificial background sky images ($\lambda_{on}$ and $\lambda_{off}$) resulting from the 2-IM method.

**Figure 5:**
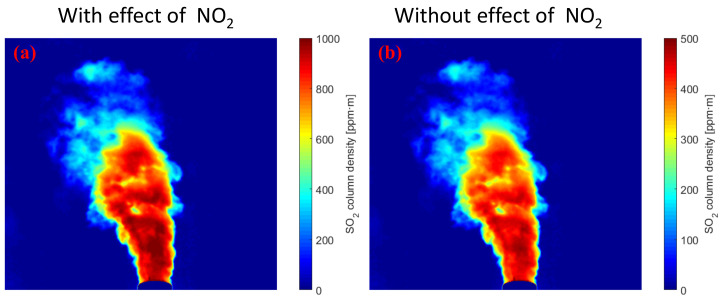
SO_2_ CD of industrial emissions obtained by a UV camera in the absence of aerosol scattering within the plume. (**a**) ignoring the effect of NO_2_ absorption; (**b**) considering the NO_2_ absorption.

The measurement accuracy of the SO_2_ CD from industrial emissions is affected by the NO_2_ absorption. Industrial emissions were measured when the plume entered the camera FOV within the plant. The camera system can be calibrated by using the real-time continuous calibration method of Wu [22,23]. In the particular case of assuming the absence of aerosols in the plume, the SO_2_ CD density from industrial emissions obtained from the real-time continuous calibration method is shown in Figure 5. Figure 5a shows the SO_2_ image ignoring the effect of NO_2_ absorption, and Figure 5b shows the SO_2_ image using the second channel to correct the NO_2_ absorption. Ignoring the NO_2_ absorption leads to an overestimation of the emissions.

### 3.2. Mie Scattering Corrections

**Figure 6:**
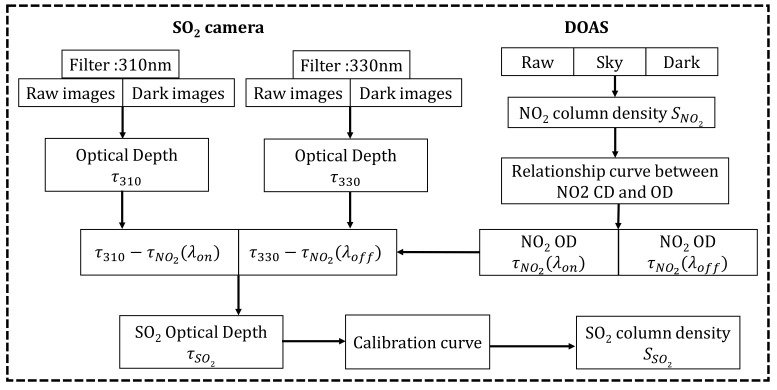
Flow chart of data analysis.

As illustrated in Figure 5, the effect of NO_2_ absorption can lead to an inaccurate SO_2_ image measurement. The result of the SO_2_ image obtained by the two methods differed twice. This finding may be the result of the NO_2_ absorption correction based on the use of a second channel. However, when scattered solar radiation reaches a camera after passing through a plume from a stack, it contains several trace gases (SO_2_ and NO_2_) and aerosol. Assuming that aerosol scattering is independent of wavelength, the second channel of the traditional camera is mostly used to correct plume aerosols in the stack. Therefore, a second channel is used to correct the aerosol or NO_2_ absorption. Although a second channel certainly reduces the influence of aerosol scattering and NO_2_ absorption on SO_2_ camera measurements, it does not completely remove it. This condition is a limitation of the SO_2_ camera. However, this problem can be overcome by correcting NO_2_ absorption and aerosol scattering. The OD of SO_2_ by applying two band-pass filters can be expressed as:

(3) $$\tau_{SO_{2}}=\tau_{310}-\tau_{330}=\left(\ln\frac{I(\lambda_{on})}{I_{0}(\lambda_{on})}-\tau_{NO_{2}}(\lambda_{on})\right)-\kappa_{Mie}\cdot \left(\ln\frac{I(\lambda_{off})}{I_{0}(\lambda_{off})}-\tau_{NO_{2}}(\lambda_{off})\right)$$

where $\kappa_{Mie}=\tau_{Mie}(\lambda_{on})/\tau_{Mie}(\lambda_{off})={\left(\lambda_{on}/\lambda_{off}\right)}^{-\alpha }$ is the ratio between the scattering cross-section at two different wavelengths $\lambda_{on}$ and $\lambda_{off}$. $\tau_{Mie}(\lambda_{on})$ and $\tau_{Mie}(\lambda_{off})$ are the plume aerosol ODs (AODs). Data analysis was conducted and is summarized in Figure 6.

**Figure 7:**
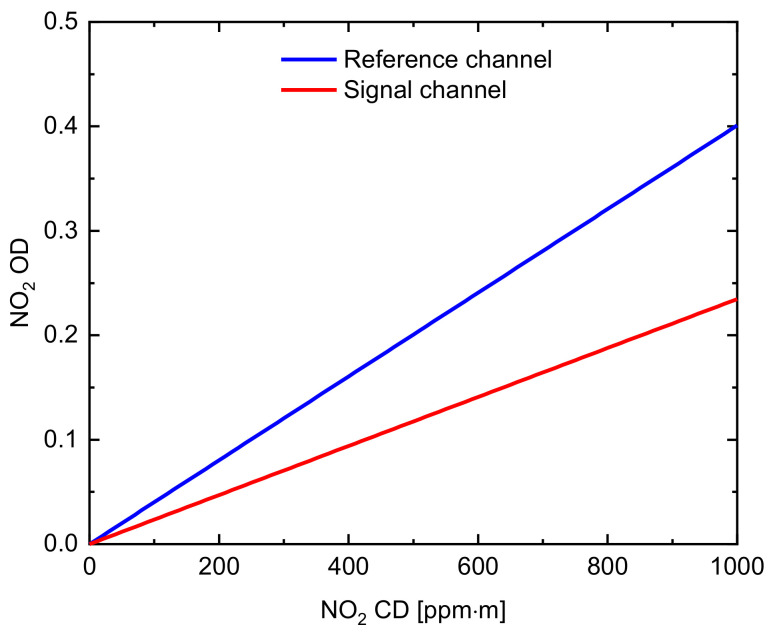
Relationship curve between the corresponding NO_2_ CD and the NO_2_ optical depth (OD).

**Figure 8:**
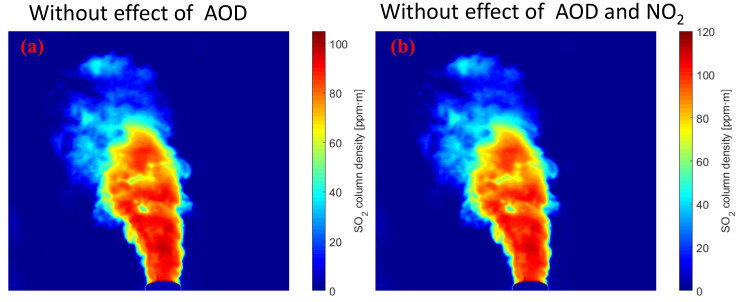
SO_2_ CD of industrial emission obtained by UV camera in the presence of aerosols within the plume. (**a**) with aerosol scattering considered; (**b**) with the NO_2_ absorption and aerosol scattering considered.

For two band-pass filters, correcting the effects of NO_2_ absorption and aerosol scattering at the same time is impossible. The corresponding signal channel ($\lambda_{on}$) and the corresponding reference channel ($\lambda_{off}$) are influenced by NO_2_ absorption and aerosol scattering. Here, the spectral data were analyzed by using DOAS to obtain the NO_2_ CD. The two cameras simultaneously capture the plume with the same FOV, and the spectrometer measures the spectral information of the plume area with the same FOV of the camera. This process was to accurately determine the area in which the DOAS optical FOV is directed and obtains the best correlation between the corresponding channel NO_2_ OD from the SO_2_ camera and the NO_2_ CD measured by DOAS. Using Equation (1), the relationship between the column density of NO_2_ and the optical density $\tau_{NO_{2}}$ is obtained. Figure 7 shows the calculated relationship curve between the signal and reference channel NO_2_ CD of the SO_2_ cameras and the NO_2_ OD. The signal channel (red line) and the reference channel (blue line) NO_2_ OD of the SO_2_ cameras are derived from the NO_2_ CD measurement by DOAS. The NO_2_ CD is converted to NO_2_ OD by multiplying with the factor obtained from the relationship curve. The subtraction of the NO_2_ OD is required to correct the NO_2_ absorption in the two channels. Figure 8b shows the concentration image of the SO_2_ plume obtained by UV cameras with the NO_2_ absorption and aerosol scattering considered. For comparison, Figure 8a shows the SO_2_ CD from UV cameras with only aerosol scattering considered. A second channel is used to correct the plume aerosols because the SO_2_ CD is underestimated because of the presence of NO_2_ absorption in the plumes. Therefore, this method using DOAS with the SO_2_ camera makes the real-time NO_2_ absorption correction. The accuracy of this method is significantly improved by the NO_2_ absorption correction.

The accuracy of the SO_2_ CD depends on the difference between the NO_2_ OD measured by DOAS and the NO_2_ OD of the corresponding channels on the SO_2_ camera. Simultaneously, it can be found that only using the second channel from the SO_2_ camera to correct for aerosol scattering results in an underestimation of the SO_2_ measurement. However, this method is practical for emission plume monitoring from industrial sources and ships, especially for SO_2_ CD measurement with NO_2_ absorption correction.

## 4. SO_2_ CD Error

The error measured by the SO_2_ camera is an extremely important quantity for accuracy. The measurement uncertainty of the SO_2_ CD can be determined through propagation of error analysis. The dominant sources of uncertainty during the measurements are the system error ($\Delta S_{1}$, uncertainty of 11.3%) and the random error ($\Delta S_{2}$, uncertainty of 7.48%). The SO_2_ CD error ΔS can be expressed as:

(4) $$\Delta S=\sqrt{{\left(\frac{\partial S}{\partial S_{1}}\cdot \Delta S_{1}\right)}^{2}+{\left(\frac{\partial S}{\partial S_{2}}\cdot \Delta S_{2}\right)}^{2}}$$

The absorption in the second channel is due to NO_2_ and aerosols. However, the correction procedure for the second channel is only used to correct for plume aerosols. The influence of NO_2_ absorption on SO_2_ camera measurements is not completely removed. The system error of the SO_2_ CD measurement is calculated under the influence of NO_2_ CD. The random error of SO_2_ CD is derived from light intensities $I(\lambda_{on})$, $I_{0}(\lambda_{on})$, $I(\lambda_{off})$, and $I_{0}(\lambda_{off})$, which are referred to as the apparent quantities. The random error of the SO_2_ OD $\Delta \tau_{SO_{2}}$ is obtained from the relation.

(5) $$\Delta \tau_{SO_{2}}=\sqrt{{\left(\frac{\partial \tau }{\partial I(\lambda_{on})}\cdot \Delta I(\lambda_{on})\right)}^{2}+{\left(\frac{\partial \tau }{\partial I_{0}(\lambda_{on})}\cdot \Delta I_{0}(\lambda_{on})\right)}^{2}+{\left(\frac{\partial \tau }{\partial I(\lambda_{off})}\cdot \Delta I(\lambda_{off})\right)}^{2}+{\left(\frac{\partial \tau }{\partial I_{0}(\lambda_{off})}\cdot \Delta I_{0}(\lambda_{off})\right)}^{2}}$$

where $\partial \tau /\partial I(\lambda_{on})$, $\partial \tau /\partial I_{0}(\lambda_{on})$, $\partial \tau /\partial I(\lambda_{off})$, and $\partial \tau /\partial I_{0}(\lambda_{off})$ represent the polarization of OD to light intensity, and ΔI is the amount of change in light intensity. The random errors of the SO_2_ OD are due to: (1) photon noise, which can be reduced by combining pixels. This precision can be improved to approximately twice by averaging 4 adjacent pixels and reducing the image size to 0.25 pixels. (2) the light dilution effect and the main source of the random error on UV SO_2_ camera measurements is related to the scattering of ambient photons [24].

**Figure 9:**
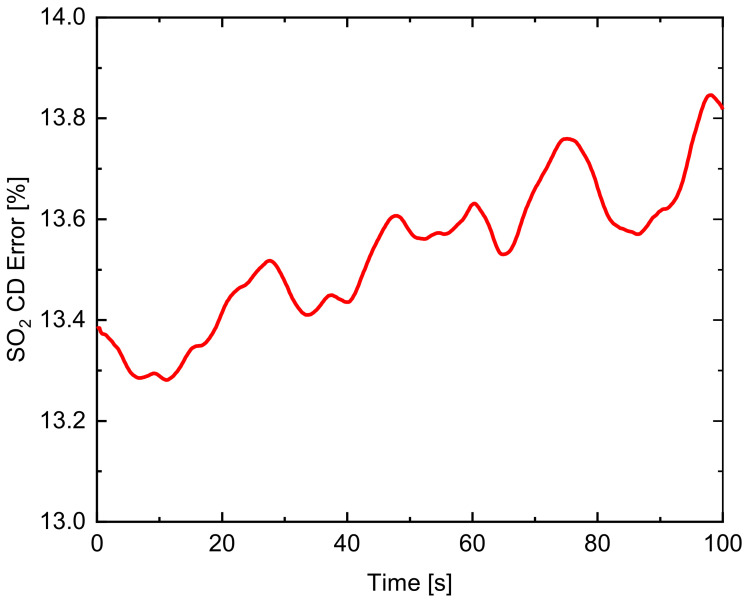
Time series of the error of the SO_2_ CD retrieved from the UV SO_2_ camera.

The time series of the error of the SO_2_ CD corrected for aerosol scattering using the second channel of the UV SO_2_ camera is shown in Figure 9. The error of the SO_2_ CD is under 14%. The variation of SO_2_ CD error is mostly due to the change of NO_2_ CD in the SO_2_ emission plume.

## 5. Conclusions

In this work, we first put forward an SO_2_ camera for the real-time NO_2_ absorption corrections to improve the measurement accuracy of SO_2_ emissions. The instrument was designed with NO_2_ absorption corrections and aerosol scattering corrections to improve the accuracy of the SO_2_ CD. The theoretical analysis for the spectral interference of industrial SO_2_ emissions is given in detail, and the error of NO_2_ absorption interference is simulated. The UV SO_2_ camera system with NO_2_ absorption corrections is developed to measure the SO_2_ CD of an industrial plume in Wuhan, and a series of experimental results are obtained to verify the accuracy of this theoretical analysis. The 2-IM method is utilized to obtain the artificial background sky images, SO_2_ CD, and the error of the SO_2_ CD. The traditional camera system only used the second channel to correct the interference of aerosol scattering, which underestimates the SO_2_ emissions due to the interference of NO_2_ absorption in the plume. We compare the experimental results with and without the effect of NO_2_ absorption correction. The SO_2_ CD errors between them are effectively reduced by 11.3% after NO_2_ absorption corrections. Therefore, an effective means to improve the accuracy of the SO_2_ CD is to combine the UV SO_2_ cameras and a spectrometer to measure the plume at the same time, which can effectively overcome the interference of NO_2_ absorption and aerosol scattering. This new method can provide a good temporal resolution for real-time NO_2_ absorption corrections, especially for measuring the plumes from industries and ships. This promising method may greatly improve the measurement accuracy of SO_2_ emissions and provide the most convenient option for rapid measurements of industrial and ship SO_2_ emissions in the foreseeable future.

## Data Availability

Not applicable.
